# Unraveling the complexities of programming neural adaptive deep brain stimulation in Parkinson’s disease

**DOI:** 10.3389/fnhum.2023.1310393

**Published:** 2023-11-28

**Authors:** Kevin B. Wilkins, Jillian A. Melbourne, Pranav Akella, Helen M. Bronte-Stewart

**Affiliations:** ^1^Department of Neurology and Neurological Sciences, Stanford University School of Medicine, Stanford, CA, United States; ^2^Department of Neurosurgery, Stanford University School of Medicine, Stanford, CA, United States

**Keywords:** Parkinson’s disease, deep brain stimulation, closed loop, adaptive, beta, subthalamic nucleus (STN), globus pallidus internus (GPi)

## Abstract

Over the past three decades, deep brain stimulation (DBS) for Parkinson’s disease (PD) has been applied in a continuous open loop fashion, unresponsive to changes in a given patient’s state or symptoms over the course of a day. Advances in recent neurostimulator technology enable the possibility for closed loop adaptive DBS (aDBS) for PD as a treatment option in the near future in which stimulation adjusts in a demand-based manner. Although aDBS offers great clinical potential for treatment of motor symptoms, it also brings with it the need for better understanding how to implement it in order to maximize its benefits. In this perspective, we outline considerations for programing several key parameters for aDBS based on our experience across several aDBS-capable research neurostimulators. At its core, aDBS hinges on successful identification of relevant biomarkers that can be measured reliably in real-time working in cohesion with a control policy that governs stimulation adaption. However, auxiliary parameters such as the window in which stimulation is allowed to adapt, as well as the rate it changes, can be just as impactful on performance and vary depending on the control policy and patient. A standardize protocol for programming aDBS will be crucial to ensuring its effective application in clinical practice.

## Introduction

Deep brain stimulation (DBS) currently offers effective treatment for motor symptoms in Parkinson’s disease (PD). However, despite its success, it still suffers from several weaknesses. These include impairment of speech, only moderate effectiveness for freezing of gait, some residual fluctuation between on/off states, and loss of efficacy over time for axial symptoms and to a lesser extent bradykinesia (Zibetti et al., 2011; Rizzone et al., 2014). Advancements in current steering, stimulation patterns, and other aspects of DBS offer the potential to improve its effectiveness for PD and other indications. One of the most promising advancements is the implementation of closed loop or adaptive DBS (aDBS) in which stimulation parameters, typically amplitude, modulate in response to a relevant biomarker (Neumann et al., 2023). The hope of such a “smart” DBS approach is to improve symptom control, lessen side effects, and potentially lessen long-term habituation.

The first aDBS work in PD in 2013 used patients with externalized leads between the first and second stage of their DBS procedure (Little et al., 2013). This work showed the initial feasibility and efficacy of an aDBS approach for PD motor symptoms. aDBS using patients with externalized leads was expanded to longer duration sessions and freely moving humans (Rosa et al., 2015; Arlotti et al., 2018), and a patient with chronic DBS (Piña-Fuentes et al., 2017). The advent of the first-generation sensing neurostimulator, Activa™ PC+S, allowed for the advancement from externalized lead patients to aDBS in chronically implanted individuals using a computer-in-the-loop system. With this device, we demonstrated safety, tolerability and efficacy of aDBS (Velisar et al., 2019), and, subsequently, demonstrated the feasibility of aDBS for freezing of gait (Petrucci et al., 2020b). The availability of the Summit^®^ RC+S for research expanded the opportunity for aDBS by both increasing its technological capabilities (e.g., biomarker selection, parameter adjustment, sampling frequency, etc.) as well as allowing implementation outside the clinic, thus allowing for aDBS at-home for the first time (O’Day et al., 2020a; Petrucci et al., 2020a; Gilron et al., 2021a; Oehrn et al., 2023). The combination of the research with Activa™ PC+S and Summit^®^ RC+S led to the Percept™ PC, which became the first commercially available DBS neurostimulator for PD to offer neural sensing capabilities (Jimenez-Shahed, 2021). The Percept™ PC also offers the capability to perform chronic aDBS in research environments. These capabilities are being tested in a pivotal international multi-site trial, which, if successful, would allow for aDBS to become a clinical option for treatment.^1^

The rapidly approaching future in which aDBS is a viable clinical option brings with it the need to better understand how it should be implemented to maximize its benefits. Just as current DBS programming has evolved to a standardized protocol, a standardization for parameter selection and evaluation for aDBS will allow the optimization of therapy. The goal of this perspective article is to offer a guide for the considerations of aDBS calibration based on our research experience across the Activa™ PC+S, Summit^®^ RC+S, and Percept™ PC (Medtronic PLC).

## Sense-friendly configuration and restrictions

**FIGURE 1:**
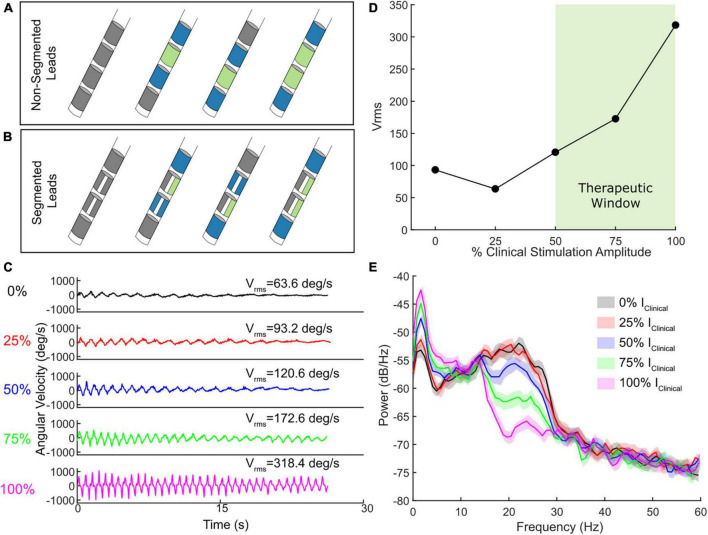
**(A)** Schematic of four contact lead sense-friendly configurations. Blue contacts represent potential recording pairs and green contacts represent stimulating contacts. **(B)** Schematic of eight contact directional lead sense-friendly configuration. Directional leads allow the ability to stimulation with one, two, or all three segments in a given row. Only one example of an active segmented contact is shown. **(C)** Example of a wrist flexion-extension task which quantitatively measures bradykinesia at different levels of stimulation intensity relevant to clinical stimulation. **(D)** The observed Vrms across the different stimulation levels, with the therapeutic window highlighted in light green. **(E)** The accompanying power spectral density plots for one STN for the wrist flexion-extension task shown in **(C)** at the different levels of stimulation intensity.

Older DBS leads had 4 cylindrical electrode contacts whereas current leads have 8 electrode contacts, 6 of which are segmented over 2 levels to allow for directional stimulation fields. One of the critical requirements for aDBS for most devices is a sense-friendly configuration that allows for recording of artifact-free local field potentials (LFPs) from the site of stimulation as a signal for aDBS. One of the main techniques for removing the stimulation artifact is through common mode rejection in which a “sandwich” is used by recording from the contacts surrounding the active stimulation contact(s) (Stanslaski et al., 2012, 2018). By taking the difference between the two recording contacts surrounding the active stimulation contact, the stimulation artifact is significantly attenuated, assuming similar impedances (Figures 1A, B). This configuration is possible if either a single contact at the second or third levels is active (single monopolar configuration) or if two contacts at the second and third levels are active (double monopolar configuration). However, this excludes certain configurations from being used for aDBS, such as those requiring the most dorsal or ventral contact. This limitation has been addressed by the AlphaDBS^®^ system which allows for asymmetrical sensing configurations (Arlotti et al., 2021).

Typically, most targeting approaches for the subthalamic nucleus (STN) or globus pallidus internus (GPi) seek to have the electrodes at the second and/or third levels placed within the target of interest, and therefore, not surprisingly, these contacts are the ones most often used as active contacts (Hamel, 2003). However, a variety of circumstances may require the use of the most dorsal or ventral contacts. Ventral contacts may be used to treat gait impairment, and dorsal contacts may be used to treat dyskinesias (Alterman et al., 2004; Herzog et al., 2007; Chastan et al., 2008; Weiss et al., 2013; Ramdhani et al., 2015). Additionally, activation of the most dorsal or ventral electrode may be required in cases of suboptimal targeting.

Adaptive deep brain stimulation (aDBS) does not necessitate that both sides have sense-friendly configurations, as aDBS can be set up in one hemisphere, while maintaining the other side on open loop DBS, or signal from one hemisphere can be used to drive aDBS in both hemispheres.

Even sense-friendly configurations can still be vulnerable to various sources of artifact that render the LFP unusable (Thenaisie et al., 2021; Hammer et al., 2022). Artifact can still be seen if there is significant mismatch in impedances between the two recording contacts surrounding the active electrode (Stanslaski et al., 2012), or if there is a mechanical issue along the extension or lead (e.g., a break, fluid intrusion, etc.). Additionally, electrocardiogram (ECG) artifact can be a source of noise, especially in lower frequencies and if the implantable pulse generator (IPG) is implanted in the left chest (Neumann et al., 2021; Thenaisie et al., 2021). LFPs are especially vulnerable to ECG artifacts during passive recharge due to an increased duration of time after each stimulation pulse in which ECG or other sources of noise can leak into the signal (Stanslaski et al., 2018). Movement, especially turning of the head or standing up, can, in some instances, elicit transient artifacts (Thenaisie et al., 2021; van Rheede et al., 2022). Lastly, ramping of stimulation is known to cause transient artifacts (Ansó et al., 2022; Hammer et al., 2022). Separating the recording and stimulation site, such as the use of cortical electrocorticography (ECoG), offers an alternative to minimize the impact of DBS-related artifacts on the signal of interest, but is not standard of care (Swann et al., 2018; Gilron et al., 2021a; Oehrn et al., 2023).

One potential future approach to avoid potential artifacts in the neural data and sensing configuration restrictions is to instead adapt stimulation based on measurements of the symptoms directly from peripheral sensors. Inertial measurement units (IMUs) with gyroscopes and accelerometers have successfully been used to detect and measure tremor, freezing of gait, bradykinesia, and dyskinesias (Griffiths et al., 2012; Braybrook et al., 2016; O’Day et al., 2020b; Powers et al., 2021). A system that enables Bluetooth communication between an external sensor and the IPG could therefore allow the ability to adapt stimulation in response to the presence or severity of various symptoms rather than relying on, or in addition to, a neural biomarker. Alternatively, sensors could be directly integrated into the IPG itself for a fully embedded system. Pilot studies have demonstrated the feasibility of this approach (Malekmohammadi et al., 2016; O’Day et al., 2020a; Cernera et al., 2021; Melbourne et al., 2023) but this capability is currently limited to research devices.

## Identifying the neural input for aDBS

There have been several approaches to selecting which signal to use as input for aDBS in PD. A relevant neural input for aDBS should ideally meet 2 specific criteria: 1, it should relate to the behavioral impairment and 2, it should be modulated by DBS in an expected and consistent manner. These two criteria ensure that the neural input is both a good biomarker of the underlying pathophysiology of the disease, and an appropriate feedback signal, so that when stimulation amplitude adjusts, it leads to corresponding changes in the biomarker.

The most commonly used neural signal for aDBS is LFP beta band (13–30 Hz) power recorded from the DBS lead, since the greater the attenuation of beta power or reduction in beta burst duration from DBS, the greater the improvement in bradykinesia, rigidity, gait impairment, and freezing of gait (FOG) (Kuhn et al., 2008; Neumann et al., 2016; Anidi et al., 2018; Kehnemouyi et al., 2021). Beta burst durations and gamma power have also been used as relevant and efficacious neural inputs, and newer methods have leaned on machine learning approaches to determine the biomarker of interest rather than *a priori* designations (Swann et al., 2018; Petrucci et al., 2020a; Gilron et al., 2021a; Merk et al., 2022; Gao et al., 2023; Oehrn et al., 2023).

Regardless of choice of biomarker, it is critical that there is sufficient signal to rely on it for aDBS. Large datasets from OR and postoperative recordings indicate the presence of beta power within the STN in a significant percentage of individuals with PD (Shreve et al., 2017; Darcy et al., 2022). The BrainSense capabilities of the Percept™ PC device allow evaluation of beta power across all contact pairs OFF DBS in chronically implanted patients to determine whether enough signal is present to use for aDBS. The choice of stimulation contact can then be chosen based on the highest observed beta power. The Percept™ PC device offers the ability to use a 5 Hz band around the frequency of maximum observed beta power (or frequency band of interest) as the input for aDBS.

Once the biomarker of interest has been established for a given patient, there are multiple approaches for the control policy. The two most common policies are a single-threshold and dual-threshold approaches (Supplementary Figure 1). In the single-threshold approach, stimulation will increase when the biomarker is above threshold, and decrease when the biomarker is below threshold (Little et al., 2013, 2016; Piña-Fuentes et al., 2020). This response to threshold can also be inverted in the case of gamma power. Whereas increased beta is often associated with greater PD impairment, increases in gamma power in the motor cortex and STN have been linked to the presence of dyskinesias and a hyperkinetic state (Swann et al., 2016; Oehrn et al., 2023), which is a sign of excessive combined therapy between DBS and medication. Therefore, stimulation decreases in response to gamma power going above threshold (Oehrn et al., 2023). Meanwhile, in a dual-threshold approach, there are two thresholds, which creates a 3rd “hold” state. In this policy, when the biomarker (e.g., beta power) is above the upper threshold stimulation increases, whereas if it is below the lower threshold, stimulation decreases, but if it is between thresholds, stimulation holds (Velisar et al., 2019).

## Determining safe and efficacious aDBS amplitude limits

Standard DBS requires determining the amplitude at which stimulation remains constant, whereas aDBS requires setting safe and efficacious minimum and maximum amplitude limits, between which aDBS varies. Most early aDBS research allowed the minimum amplitude to be at zero (Little et al., 2013, 2016; Velisar et al., 2019; Piña-Fuentes et al., 2020; He et al., 2023). However, as the research into the efficacy of aDBS has evolved, it is now evident that setting a non-zero, therapeutically acceptable, minimum limit for aDBS amplitude can protect against lowering aDBS amplitude to sub-therapeutic levels, which may result PD symptom return, especially tremor (Velisar et al., 2019; He et al., 2023). The upper stimulation amplitude is that above which there may be side effects due to over stimulation such as dyskinesias, face pulling, speech intelligibility, or paresthesias. Accurate determination of these limits is crucial for ensuring therapeutic level of stimulation during aDBS.

The growing accessibility of wearables (e.g., IMUs) and measurement devices offers the opportunity to determine safe and efficacious lower and upper limits of stimulation. For instance, angular velocity, accelerometry, and other forms of behavioral data can be used to provide high-resolution quantitative metrics of behaviors and symptoms to aid clinicians evaluating the therapeutic range of DBS in a patient. Amplitude titrations where behavior is assessed at randomized presentations of various intensities can provide a clear picture for patient-specific lower and upper limits for aDBS with a therapeutic response (i.e., therapeutic window). For instance, an instrumented assessment of bradykinesia with DBS titrations can allow for the identification of the minimum level of stimulation that provides therapeutic benefit (Figures 1C, D). These titrations can also be used for assessment of other symptoms such as tremor or gait impairment (Supplementary Figure 2). Combining DBS titrations with high-resolution wearables and/or measurement devices that provide digestible reports of behavior in near real time can both automate and reduce the time needed for determining stimulation limits for aDBS.

Deep brain stimulation (DBS) titrations offer the additional benefit of evaluating how the neural biomarker of interest responds to DBS within the aDBS amplitude limits (Figure 1E). Although it is important to identify the presence of a biomarker (e.g., beta power) OFF DBS, it is important to confirm that it also modulates with incremental adjustments of DBS. Both single and dual threshold control policies hinge on the assumption that beta (or the frequency band power of interest) modulates in an expected manner. Therefore, one must ensure both that the band chosen is not inert and that there is sufficient modulation specifically within the aDBS limits (i.e., I_min_ to I_max_) as that is where stimulation will be during aDBS. If not, then aDBS will simply be adapting primarily on noise, rather than the pathophysiological marker. This modulation should be relatively continuous in fashion if stimulation amplitude is adapted in a graded manner. An alternative viable approach is if the biomarker modulates in a binary fashion (e.g., present of absent) when combined with adapting between two stimulation states (e.g., lower level and higher level of stimulation amplitude).

The inverse relationship between beta power and DBS amplitude enables the choice of beta power thresholds directly from the choice of I_min_ and I_max_. For the dual threshold algorithm, the upper beta threshold is set at the beta power that is measured during DBS at I_min_; the lower beta threshold is either that measured during DBS at I_max_ or a value midway between beta power at I_min_ and that at I_max_ (Velisar et al., 2019). The beta threshold for single threshold aDBS has been traditionally chosen as 75% of beta power OFF DBS. Arbitrarily deciding these thresholds independent of how the neural biomarker responds to DBS amplitude may lead to suboptimal adaptation. Medication further complicates the decision of thresholds since medication also attenuates beta. Typically, thresholds are identified first off medication, and then altered, if necessary, when tested on medication. This may involve lowering the beta threshold due to medication-induced reductions in overall beta power. Typically streaming the data during aDBS setup allows confirmation of whether there is sufficient modulation with aDBS or if lowering of the beta threshold is required, as would be the case if stimulation amplitude plummeted to I_min_ in the presence of medication.

Although these biomarkers are typically evaluated in clinic, many of these signals vary with activity and over the course of the day and night (Fleming et al., 2022; van Rheede et al., 2022; Feldmann et al., 2023). Therefore, future work may establish the validity of combining these in-clinic recordings with extended at-home recordings to find the biomarker of interest, and understand its relation to, activity, sleep and circadian rhythms (Toth et al., 2020; Gilron et al., 2021b; Smyth et al., 2023).

## Ramp rate evaluation

After establishing the biomarker, control policy and thresholds for aDBS, and window to allow stimulation to adjust within, the last critical decision is how fast or slow to allow stimulation to change (i.e., ramp rate). Determining the ramp rate is both goal- and patient-specific. The ramp rate and control policy used go hand-in-hand. For instance, single-threshold control policies are often combined with rapid ramp rates to quickly provide maximum amount of stimulation to respond to the biomarker. Typically, these rapid rates attempt to traverse from I_min_ to I_max_ in roughly 250 ms (e.g., 8 mA/s if traversing a 2 mA range) (Little et al., 2013). When using this rapid ramp rate, initial single-threshold policies typically fluctuated between 0 mA and I_max_ with a goal of stimulation being active roughly 50% of the time. However, it may be beneficial to use an I_min_ rather than dropping stimulation all the way to 0, as discussed earlier. Dual-threshold control policies are often used for slow time courses, such as adjusting stimulation based on medication-induced fluctuations which occur on the order of minutes to tens of minutes) or intermediate time courses on the order of seconds (0.1–0.25 mA/s) (Velisar et al., 2019). Sometimes faster ramp ups in comparison to ramp down (e.g., 2× faster) are implemented to bias toward higher therapy and protect against stimulation dropping too fast.

**FIGURE 2:**
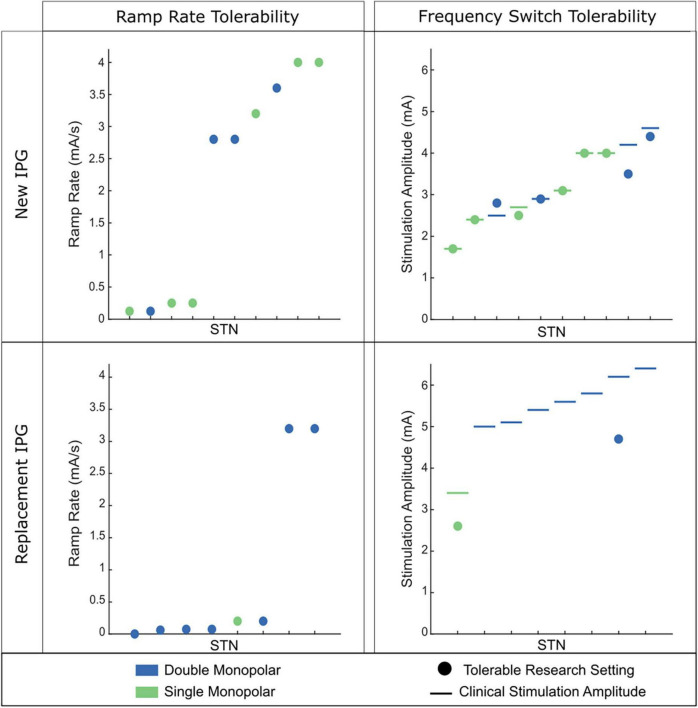
Tolerability of ramp rate across 18 STNs (9 individuals). **(Left)** Observed tolerability for ramp rate of stimulation intensity. **(Right)** Observed tolerability of instantaneous frequency switching between 140 and 60 Hz. The participant’s clinical stimulation is represented by the horizontal line. The circle depicts the tolerable amplitude found for frequency switching. If no circle is present, no tolerable therapeutic amplitude was found for frequency switching. **(Top)** Patient cohort who are on their first IPG. **(Bottom)** Patient cohort who have had at least one IPG replacement. Green dots indicate the patient had a single monopolar stimulation configuration and blue dots indicate a double monopolar stimulation configuration. Patients on their first IPG showed better tolerability of faster ramp rates of intensity and higher amplitudes for frequency switching. Patients on replacement IPGs rarely tolerated fast ramp rates of intensity or instantaneous frequency switching.

Although one may have a target ramp rate to use based on the specific goal of aDBS, the ramp rate used is patient-specific as there is large variability among what is tolerated. Ramping too fast can elicit symptoms such as paresthesia or nausea that may make aDBS intolerable. These symptoms tend to be most prevalent when stimulation is increasing and at the top of the stimulation range (Petrucci et al., 2021). The observed variability in patient-tolerability may be due to a host of factors, including max amplitude of stimulation, size of the range that stimulation is traversing, number of contacts active (e.g., single vs. double monopolar), electrode location within the target, impairment level and disease severity, and how long a patient has been on DBS (Koeglsperger et al., 2019). In our experience, less impaired patients on their first DBS IPG tend to be much more tolerable to rapid ramp rates (Figure 2). Meanwhile, more impaired patients who have been on DBS much longer (e.g., have had one or multiple IPG replacements), require higher amplitudes, and often double monopolar configurations tend to not be able to tolerate fast ramp rates or, in some instances, any ramping at all. Similar variability was observed when altering different stimulation parameters, such as instantaneous frequency switching between 140 and 60 Hz (Figure 2).

Despite the variability in ramp rate tolerability among patients, there are several approaches that can be used to try and achieve tolerability. These include lowering I_max_ and/or slowing down the rate. The decision of which of these two approaches to take will depend on the goal of the aDBS and the patient. For more impaired individuals, lowering I_max_ may not be a viable option as the patient needs more stimulation for therapeutic benefit. Therefore, the main option would be to simply slow down the ramp rate until achieving tolerability (and perhaps only adapt one side if it is found that only one side is contributing to the side effects). Meanwhile, in a less impaired patient lowering I_max_ may prove more beneficial if it means a more rapid ramp rate can be implemented that can get the person on high levels of stimulation quicker.

## Potential shortcomings, solutions, and alternative approaches

The growing availability of aDBS offers exciting promise for the therapeutic potential of DBS, but there are several potential shortcomings. Although beta power has been identified as a potential useful biomarker for aDBS due to its relation to bradykinesia and rigidity alongside its responsiveness to DBS and medication, it may not be a suitable for everyone. Tremor and voluntary movement have both been shown to attenuate beta, which may lead to unwanted decreases in stimulation amplitude (Quinn et al., 2015; Shreve et al., 2017; Velisar et al., 2019; Eisinger et al., 2020). However, there are potential workarounds to combat this. For instance, ensuring that the lower stimulation limit (i.e., I_min_) is set at a high enough stimulation amplitude where tremor is sufficiently controlled can ensure that stimulation will maintain therapeutic efficacy for tremor even with drops in beta power. Similarly, one can protect against voluntary movement-related attenuation in beta by requiring a sufficient onset duration (i.e., the amount of time required for the biomarker to be above or below threshold before making a stimulation decision). Reduction in beta power is most strongly association with the initiation of movement, so onset durations > 1 s can ensure transient movement-related reductions in beta do not lead to inadvertent decreases in stimulation. A potential long-term solution to both problems is the identification of alternative biomarkers, but this may be challenging with the current hardware limitations.

It is important to recognize that aDBS is just one of several possible approaches to deal with some of the limitations of current DBS. Side effects from DBS may also be avoided with directional current steering, which allows more efficient targeting of the region of interest without spillover to neighboring regions that elicit common side effects such as dysarthria (Timmermann et al., 2015; Dembek et al., 2017; Umemura et al., 2023). Similarly, more precise understanding of the anatomy through high-resolution imaging can enable identification of sweet spots for different symptoms within the STN or GPi (Hilliard et al., 2011; Dembek et al., 2019; Horn, 2019). Coupling this anatomy with directional steering may allow for better treatment for refractory symptoms such as freezing of gait. Alternative stimulation approaches, such as coordinated reset or theta burst stimulation (Wang et al., 2016; Horn et al., 2020), may also provide a different approach for treatment refractory symptoms. These approaches can be implemented independently or combined with aDBS. For instance, current directional leads offer the potential to combine current steering with aDBS, but it is still too early to know how feasible this approach may be.

## Conclusion

aDBS offers an exciting advancement for current DBS technologies. Although, it will require tuning more parameters than traditional continuous open loop DBS, a standard and logical protocol makes aDBS set up straightforward. The initial choice of the active electrodes is made simpler than the previous method of monopolar review by the capability to sense beta power from all the available electrodes: substantial evidence has shown that the active electrode that is closest to the site of maximum beta power has the best therapeutic outcome. In this Perspective we have outlined several of the key considerations for implementing aDBS based on our research and clinical experience with the Activa™ PC+S, Summit^®^ RC+S, and Percept™ PC.

Implementing aDBS first requires a sense-friendly stimulation configuration and the presence of a viable biomarker to serve as the neural input for aDBS. A relevant neural input should both relate the behavioral impairment and modulate with DBS amplitude in an expected and consistent manner. The most popular neural input to date for PD is beta power due to its presence in PD, relation to impairment levels, and modulation by DBS intensity. In addition to the neural input, accurate determination of the window in which stimulation will adjust within is crucial for maintaining acceptable therapeutic efficacy with aDBS. Ideally this window should be determined quantitatively with either wearable sensors or measurement devices that can accurately show the lower and upper limits of stimulation that provide therapeutic benefit without adverse side effects but this can also be done using clinical assessment. The establishment of the lower and upper limits should go hand in hand with the thresholds that are used for the neural control policy, as aDBS will be modulating within the defined amplitude window based on the observed biomarker.

The goal of aDBS will impact both the control policy choice (e.g., single- or dual-threshold) as well as the rate as which stimulation amplitude should adjust. It is worth noting that the field is still in the early stages of understanding how tuning of these various parameters impacts overall performance. The development of optimization strategies for simplifying these decisions and understanding which parameters most impact overall performance will be critical for wide-level successful adoption. Additionally, future devices may expand this space even further as aDBS increases in sophistication, such as developing to adapt other stimulation parameters besides amplitude (e.g., frequency, pulse width, active contact, etc.), respond to more sophisticated neural biomarkers besides just power in a frequency band as well as access to higher frequencies, and ability to run aDBS based on other non-neural signals such as those from peripheral sensors or embedded sensors in the IPG itself.

## Data availability statement

The raw data supporting the conclusions of this article will be made available by the authors, without undue reservation.

## Ethics statement

The studies involving humans were approved by the Stanford Institutional Review Board. The studies were conducted in accordance with the local legislation and institutional requirements. The participants provided their written informed consent to participate in this study.

## Author contributions

KW: Conceptualization, Formal analysis, Investigation, Visualization, Writing – original draft, Writing – review & editing. JM: Conceptualization, Formal analysis, Investigation, Visualization, Writing – original draft, Writing – review & editing. PA: Conceptualization, Data curation, Formal analysis, Writing – review & editing. HB-S: Conceptualization, Funding acquisition, Supervision, Writing – original draft, Writing – review & editing.

## Footnotes

1
https://clinicaltrials.gov/ct2/show/NCT04547712

## References

[B1] Alterman R. Shils J. Gudesblatt M. Tagliati M. (2004). Immediate and sustained relief of levodopa-induced dyskinesias after dorsal relocation of a deep brain stimulation lead: Case report. *FOC* 17 39–42. 10.3171/foc.2004.17.1.6 15264775

[B2] Anidi C. O’Day J. Anderson R. Afzal M. Syrkin-Nikolau J. Velisar A. (2018). Neuromodulation targets pathological not physiological beta bursts during gait in Parkinson’s disease. *Neurobiol. Dis.* 120 107–117. 30196050 10.1016/j.nbd.2018.09.004 PMC6422345

[B3] Ansó J. Benjaber M. Parks B. Parker S. Oehrn C. Petrucci M. (2022). Concurrent stimulation and sensing in bi-directional brain interfaces: A multi-site translational experience. *J. Neural Eng.* 19:026025. 10.1088/1741-2552/ac59a3 35234664 PMC9095704

[B4] Arlotti M. Colombo M. Bonfanti A. Mandat T. Lanotte M. Pirola E. (2021). A new implantable closed-loop clinical neural interface: First application in Parkinson’s Disease. *Front. Neurosci.* 15:763235. 10.3389/fnins.2021.763235 34949982 PMC8689059

[B5] Arlotti M. Marceglia S. Foffani G. Volkmann J. Lozano A. Moro E. (2018). Eight-hours adaptive deep brain stimulation in patients with Parkinson disease. *Neurology* 90 e971–e976. 29444973 10.1212/WNL.0000000000005121 PMC5858949

[B6] Braybrook M. O’Connor S. Churchward P. Perera T. Farzanehfar P. Horne M. (2016). An ambulatory tremor score for Parkinson’s Disease. *J. Parkinson’s Dis.* 6 723–731. 27589540 10.3233/JPD-160898

[B7] Cernera S. Alcantara J. Opri E. Cagle J. Eisinger R. Boogaart Z. (2021). Wearable sensor-driven responsive deep brain stimulation for essential tremor. *Brain Stimul.* 14 1434–1443. 10.1016/j.brs.2021.09.002 34547503

[B8] Chastan N. Westby G. Yelnik J. Bardinet E. Do M. Agid Y. (2008). Effects of nigral stimulation on locomotion and postural stability in patients with Parkinson’s disease. *Brain* 132 172–184. 10.1093/brain/awn294 19001482

[B9] Darcy N. Lofredi R. Al-Fatly B. Neumann W. Hübl J. Brücke C. (2022). Spectral and spatial distribution of subthalamic beta peak activity in Parkinson’s disease patients. *Exp. Neurol.* 356:114150. 10.1016/j.expneurol.2022.114150 35732220

[B10] Dembek T. Reker P. Visser-Vandewalle V. Wirths J. Treuer H. Klehr M. (2017). Directional DBS increases side-effect thresholds—A prospective, double-blind trial. *Mov. Disord.* 32 1380–1388. 10.1002/mds.27093 28843009

[B11] Dembek T. Roediger J. Horn A. Reker P. Oehrn C. Dafsari H. (2019). Probabilistic sweet spots predict motor outcome for deep braian stimulation in Parkinson disease. *Ann. Neurol.* 86 527–538. 10.1002/ana.25567 31376171

[B12] Eisinger R. Cagle J. Opri E. Alcantara J. Cernera S. Foote K. (2020). Parkinsonian beta dynamics during rest and movement in the dorsal pallidum and subthalamic nucleus. *J. Neurosci.* 40 2859–2867. 10.1523/JNEUROSCI.2113-19.2020 32107277 PMC7117906

[B13] Feldmann L. Lofredi R. Al-Fatly B. Busch J. Mathiopoulou V. Roediger J. (2023). Christmas-related reduction in beta activity in Parkinson’s Disease. *Mov. Disord.* 38 692–697. 10.1002/mds.29334 36718788

[B14] Fleming J. Kremen V. Gilron R. Gregg N. Zamora M. Dijk D. (2022). Embedding digital chronotherapy into bioelectronic medicines. *iScience* 25:104028. 10.1016/j.isci.2022.104028 35313697 PMC8933700

[B15] Gao Q. Schimdt S. Chowdhury A. Feng G. Peters J. Genty K. (2023). Offline learning of closed-loop deep brain stimulation controllers for Parkinson Disease treatment. *arXiv[Preprint].* Available online at: https://arxiv.org/abs/2302.02477 (accessed September 12, 2023).

[B16] Gilron R. Little S. Perrone R. Wilt R. de Hemptinne C. Yaroshinsky M. (2021a). Long-term wireless streaming of neural recordings for circuit discovery and adaptive stimulation in individuals with Parkinson’s disease. *Nat. Biotechnol.* 39 1078–1085. 10.1038/s41587-021-00897-5 33941932 PMC8434942

[B17] Gilron R. Little S. Wilt R. Perrone R. Anso J. Starr P. (2021b). Sleep-aware adaptive deep brain stimulation control: Chronic use at home with dual independent linear discriminate detectors. *Front. Neurosci.* 15:732499. 10.3389/fnins.2021.732499. 34733132 PMC8558614

[B18] Griffiths R. Kotschet K. Arfon S. Xu Z. Johnson W. Drago J. (2012). Automated assessment of bradykinesia and dyskinesia in Parkinson’s disease. *J. Parkinson’s Dis.* 2 47–55. 23939408 10.3233/JPD-2012-11071

[B19] Hamel W. (2003). Deep brain stimulation of the subthalamic nucleus in Parkinson’s disease: Evaluation of active electrode contacts. *J. Neurol. Neurosurg. Psychiatry* 74 1036–1046. 12876231 10.1136/jnnp.74.8.1036 PMC1738607

[B20] Hammer L. Kochanski R. Starr P. Little S. (2022). Artifact characterization and a multipurpose template-based offline removal solution for a sensing-enabled deep brain stimulation device. *Stereotact. Funct. Neurosurg.* 100 168–183. 10.1159/000521431 35130555 PMC9064887

[B21] He S. Baig F. Merla A. Torrecillos F. Perera A. Wiest C. (2023). Beta-triggered adaptive deep brain stimulation during reaching movement in Parkinson’s disease. *Brain* [Online ahead of print]. 10.1093/brain/awad233 37433037 PMC10690014

[B22] Herzog J. Pinsker M. Wasner M. Steigerwald F. Wailke S. Deuschl G. (2007). Stimulation of subthalamic fibre tracts reduces dyskinesias in STN-DBS. *Mov. Disord.* 22 679–684. 10.1002/mds.21387 17266046

[B23] Hilliard J. Frysinger R. Elias W. (2011). Effective subthalamic nucleus deep brain stimulation sites may differ for tremor, bradykinesia and gait disturbances in Parkinson’s Disease. *Stereotact. Funct. Neurosurg.* 89 357–364. 10.1159/000331269 22104373

[B24] Horn A. (2019). The impact of modern-day neuroimaging on the field of deep brain stimulation. *Curr. Opin. Neurol.* 32 511–520. 10.1097/WCO.0000000000000679 30844863

[B25] Horn M. Gulberti A. Gülke E. Buhmann C. Gerloff C. Moll C. (2020). A new stimulation mode for deep brain stimulation in Parkinson’s Disease: Theta burst stimulation. *Mov. Disord.* 35 1471–1475. 32357269 10.1002/mds.28083

[B26] Jimenez-Shahed J. (2021). Device profile of the percept PC deep brain stimulation system for the treatment of Parkinson’s disease and related disorders. *Expert Rev. Med. Dev.* 18 319–332. 10.1080/17434440.2021.1909471 33765395

[B27] Kehnemouyi Y. Wilkins K. Anidi C. Anderson R. Afzal M. Bronte-Stewart H. (2021). Modulation of beta bursts in subthalamic sensorimotor circuits predicts improvement in bradykinesia. *Brain* 144 473–486. 10.1093/brain/awaa394 33301569 PMC8240742

[B28] Koeglsperger T. Palleis C. Hell F. Mehrkens J. Bötzel K. (2019). Deep brain stimulation programming for movement disorders: Current concepts and evidence-based strategies. *Front. Neurol.* 10:410. 10.3389/fneur.2019.00410 31231293 PMC6558426

[B29] Kuhn A. Kempf F. Brucke C. Gaynor Doyle L. Martinez-Torres I. Pogosyan A. (2008). High-frequency stimulation of the subthalamic nucleus suppresses oscillatory activity in patients with Parkinson’s Disease in parallel with improvement in motor performance. *J. Neurosci.* 28 6165–6173. 10.1523/JNEUROSCI.0282-08.2008 18550758 PMC6670522

[B30] Little S. Pogosyan A. Neal S. Zavala B. Zrinzo L. Hariz M. (2013). Adaptive deep brain stimulation in advanced Parkinson disease. *Ann. Neurol.* 74 449–457. 23852650 10.1002/ana.23951 PMC3886292

[B31] Little S. Tripoliti E. Beudel M. Pogosyan A. Cagnan H. Herz D. (2016). Adaptive deep brain stimulation for Parkinson’s disease demonstrates reduced speech side effects compared to conventional stimulation in the acute setting. *J. Neurol. Neurosurg. Psychiatry* 87 1388–1389. 10.1136/jnnp-2016-313518 27530809 PMC5136720

[B32] Malekmohammadi M. Herron J. Velisar A. Blumenfeld Z. Trager M. Chizeck H. (2016). Kinematic adaptive deep brain stimulation for resting tremor in Parkinson’s Disease. *Mov. Disord.* 31 426–428. 26813875 10.1002/mds.26482

[B33] Melbourne J. Kehnemouyi Y. O’Day J. Wilkins K. Gala A. Petrucci M. (2023). Kinematic adaptive deep brain stimulation for gait impairment and freezing of gait in Parkinson’s disease. *Brain Stimul.* 16 1099–1101. 10.1016/j.brs.2023.07.003 37429355 PMC12673813

[B34] Merk T. Peterson V. Lipski W. Blankertz B. Turner R. Li N. (2022). Electrocorticography is superior to subthalamic local field potentials for movement decoding in Parkinson’s disease. *eLife* 11:e75126. 10.7554/eLife.75126 35621994 PMC9142148

[B35] Neumann W. Degen K. Schneider G. Brücke C. Huebl J. Brown P. (2016). Subthalamic synchronized oscillatory activity correlates with motor impairment in patients with Parkinson’s disease. *Mov. Disord.* 31 1748–1751. 27548068 10.1002/mds.26759 PMC5120686

[B36] Neumann W. Gilron R. Little S. Tinkhauser G. (2023). Adaptive deep brain stimulation: From experimental evidence toward practical implementation. *Mov. Disord.* 38 937–948. 10.1002/mds.29415 37148553

[B37] Neumann W. Memarian Sorkhabi M. Benjaber M. Feldmann L. Saryyeva A. Krauss J. (2021). The sensitivity of ECG contamination to surgical implantation site in brain computer interfaces. *Brain Stimul.* 14 1301–1306. 10.1016/j.brs.2021.08.016 34428554 PMC8460992

[B38] O’Day J. Kehnemouyi Y. Petrucci M. Anderson R. Herron J. Bronte-Stewart H. (2020a). “Demonstration of kinematic-based closed-loop deep brain stimulation for mitigating freezing of gait in people with Parkinson’s Disease,” in *Proceedings of the Annual International Conference of the IEEE Engineering in Medicine and Biology Society, EMBS*, (Orlando, FL: IEEE), 3612–3616. 10.1109/EMBC44109.2020.9176638 PMC8191492 33018784

[B39] O’Day J. Syrkin-Nikolau J. Anidi C. Kidzinski L. Delp S. Bronte-Stewart H. (2020b). The turning and barrier course reveals gait parameters for detecting freezing of gait and measuring the efficacy of deep brain stimulation. *PLoS One* 15:e0231984. 10.1371/journal.pone.0231984 32348346 PMC7190141

[B40] Oehrn C. Cernera S. Hammer L. Shcherbakova M. Yao J. Hahn A. (2023). Personalized chronic adaptive deep brain stimulation outperforms conventional stimulation in Parkinson’s disease. *medRXiv[Preprint].* Available online at: http://medrxiv.org/lookup/doi/10.1101/2023.08.03.23293450 (accessed September 12, 2023). 10.1038/s41591-024-03196-z PMC11826929 39160351

[B41] Petrucci M. Anderson R. O’Day J. Kehnemouyi Y. Herron J. Bronte-Stewart H. (2020a). “A closed-loop deep brain stimulation approach for mitigating burst durations in people with Parkinson’s Disease,” in *Proceedings of the Annual International Conference of the IEEE Engineering in Medicine and Biology Society, EMBS*, (New Orleans, LA: IEEE), 3617–3620. 10.1109/EMBC44109.2020.9176196 PMC8212866 33018785

[B42] Petrucci M. Neuville R. Afzal M. Velisar A. Anidi C. Anderson R. (2020b). Neural closed-loop deep brain stimulation for freezing of gait. *Brain Stimul.* 13 1320–1322. 32634599 10.1016/j.brs.2020.06.018 PMC8189032

[B43] Petrucci M. Wilkins K. Orthlieb G. Kehnemouyi Y. O’Day J. Herron J. (2021). “Ramp rate evaluation and configuration for safe and tolerable closed-loop deep brain stimulation,” in *Proceedings of the 2021 10th International IEEE/EMBS Conference on Neural Engineering (NER) [Internet]*, (Italy: IEEE). 10.1109/ner49283.2021.9441336 PMC9097241 35574294

[B44] Piña-Fuentes D. Little S. Oterdoom M. Neal S. Pogosyan A. Tijssen M. (2017). Adaptive DBS in a Parkinson’s patient with chronically implanted DBS: A proof of principle. *Mov. Disord.* 32 1253–1254. 10.1002/mds.26959 28589687 PMC5580798

[B45] Piña-Fuentes D. van Dijk J. van Zijl J. Moes H. van Laar T. Oterdoom D. (2020). Acute effects of adaptive Deep Brain Stimulation in Parkinson’s disease. *Brain Stimul.* 13 1507–1516. 32738409 10.1016/j.brs.2020.07.016 PMC7116216

[B46] Powers R. Etezadi-Amoli M. Arnold E. Kianian S. Mance I. Gibiansky M. (2021). Smartwatch inertial sensors continuously monitor real-world motor fluctuations in Parkinson’s disease. *Sci. Transl. Med.* 13:eabd7865. 10.1126/scitranslmed.abd7865 33536284

[B47] Quinn E. Blumenfeld Z. Velisar A. Koop M. Shreve L. Trager M. (2015). Beta oscillations in freely moving Parkinson’s subjects are attenuated during deep brain stimulation. *Mov. Disord.* 30 1750–1758. 26360123 10.1002/mds.26376

[B48] Ramdhani R. Patel A. Swope D. Kopell B. (2015). Early use of 60 Hz frequency subthalamic stimulation in Parkinson’s Disease: A case series and review. *Neuromodulation* 18 664–669. 10.1111/ner.12288 25833008

[B49] Rizzone M. Fasano A. Daniele A. Zibetti M. Merola A. Rizzi L. (2014). Long-term outcome of subthalamic nucleus DBS in Parkinson’s disease: From the advanced phase towards the late stage of the disease? *Parkinsonism Relat. Disord.* 20 376–381. 10.1016/j.parkreldis.2014.01.012 24508574

[B50] Rosa M. Arlotti M. Ardolino G. Cogiamanian F. Marceglia S. Di Fonzo A. (2015). Adaptive deep brain stimulation in a freely moving Parkinsonian patient. *Mov. Disord.* 30 1003–1005. 10.1002/mds.26241 25999288 PMC5032989

[B51] Shreve L. Velisar A. Malekmohammadi M. Koop M. Trager M. Quinn E. (2017). Subthalamic oscillations and phase amplitude coupling are greater in the more affected hemisphere in Parkinson’s disease. *Clin. Neurophysiol.* 128 128–137. 10.1016/j.clinph.2016.10.095 27889627

[B52] Smyth C. Anjum M. Ravi S. Denison T. Starr P. Little S. (2023). Adaptive Deep Brain Stimulation for sleep stage targeting in Parkinson’s disease. *Brain Stimul.* 16 1292–1296. 37567463 10.1016/j.brs.2023.08.006 PMC10835741

[B53] Stanslaski S. Afshar P. Cong P. Giftakis J. Stypulkowski P. Carlson D. (2012). Design and validation of a fully implantable, chronic, closed-loop neuromodulation device with concurrent sensing and stimulation. *IEEE Trans. Neural Syst. Rehabil. Eng.* 20 410–421. 10.1109/TNSRE.2012.2183617 22275720

[B54] Stanslaski S. Herron J. Chouinard T. Bourget D. Isaacson B. Kremen V. (2018). A chronically implantable neural coprocessor for investigating the treatment of neurological disorders. *IEEE Trans. Biomed. Circuits Syst.* 12 1230–1245. 10.1109/TBCAS.2018.2880148 30418885 PMC6415546

[B55] Swann N. De Hemptinne C. Miocinovic S. Qasim S. Wang S. Ziman N. (2016). Gamma oscillations in the hyperkinetic state detected with chronic human brain recordings in Parkinson’s disease. *J. Neurosci.* 36 6445–6458. 10.1523/JNEUROSCI.1128-16.2016 27307233 PMC5015781

[B56] Swann N. de Hemptinne C. Thompson M. Miocinovic S. Miller A. Gilron R. (2018). Adaptive deep brain stimulation for Parkinson’s disease using motor cortex sensing. *J. Neural Eng.* 15:046006. 10.1088/1741-2552/aabc9b PMC6021210 29741160

[B57] Thenaisie Y. Palmisano C. Canessa A. Keulen B. Capetian P. Jiménez M. (2021). Towards adaptive deep brain stimulation: Clinical and technical notes on a novel commercial device for chronic brain sensing. *J. Neural Eng.* 18:042002. 10.1088/1741-2552/ac1d5b 34388744

[B58] Timmermann L. Jain R. Chen L. Maarouf M. Barbe M. Allert N. (2015). Multiple-source current steering in subthalamic nucleus deep brain stimulation for Parkinson’s disease (the VANTAGE study): A non-randomised, prospective, multicentre, open-label study. *Lancet Neurol.* 14 693–701. 10.1016/S1474-4422(15)00087-3 26027940

[B59] Toth R. Zamora M. Ottaway J. Gillbe T. Martin S. Benjaber M. (2020). “DyNeuMo Mk-2: An investigational circadian-locked neuromodulator with responsive stimulation for applied chronobiology,” in *Proceedings of the 2020 IEEE International Conference on Systems, Man, and Cybernetics (SMC) [Internet]*, (Toronto, ON: IEEE). 10.1109/SMC42975.2020.9283187 PMC7116879 33692611

[B60] Umemura A. Oyama G. Iwamuro H. Shimo Y. Hatano T. Kamo H. (2023). Application of current steering with MICC directional lead in STN-DBS for Parkinson’s disease. *Deep Brain Stimul.* 1 20–25.

[B61] van Rheede J. Feldmann L. Busch J. Fleming J. Mathiopoulou V. Denison T. (2022). Diurnal modulation of subthalamic beta oscillatory power in Parkinson’s disease patients during deep brain stimulation. *Npj Parkinsons Dis.* 8:88. 10.1038/s41531-022-00350-7 35804160 PMC9270436

[B62] Velisar A. Syrkin-Nikolau J. Blumenfeld Z. Trager M. Afzal M. Prabhakar V. (2019). Dual threshold neural closed loop deep brain stimulation in Parkinson disease patients. *Brain Stimul.* 12 868–876. 30833216 10.1016/j.brs.2019.02.020

[B63] Wang J. Nebeck S. Muralidharan A. Johnson M. Vitek J. Baker K. (2016). Coordinated reset deep brain stimulation of subthalamic nucleus produces long-lasting, dose-dependent motor improvements in the 1-Methyl-4-phenyl-1,2,3,6-tetrahydropyridine Non-human primate model of Parkinsonism. *Brain Stimul.* 9 609–617. 10.1016/j.brs.2016.03.014 27151601 PMC10226766

[B64] Weiss D. Walach M. Meisner C. Fritz M. Scholten M. Breit S. (2013). Nigral stimulation for resistant axial motor impairment in Parkinson’s disease? A randomized controlled trial. *Brain* 136 2098–2108. 10.1093/brain/awt122 23757762 PMC3692032

[B65] Zibetti M. Merola A. Rizzi L. Ricchi V. Angrisano S. Azzaro C. (2011). Beyond nine years of continuous subthalamic nucleus deep brain stimulation in Parkinson’s disease: 9 Years of STN-DBS in PD. *Mov. Disord.* 26 2327–2334. 10.1002/mds.23903 22012750

