# Supplementary material for: Unraveling the complexities of programming neural adaptive deep brain stimulation in Parkinson’s disease
Source: Front Hum Neurosci. 2023 Nov 28;17:1310393. doi: 10.3389/fnhum.2023.1310393 (PMC10716917; doi:10.3389/fnhum.2023.1310393)
Supplement: Supplementary file 1 [file Data_Sheet_1.docx]

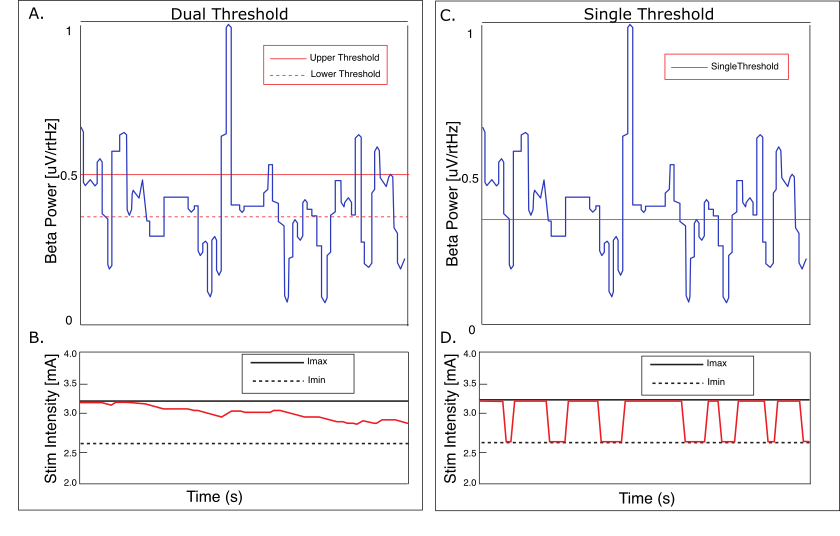


Supplementary Figure 1. Schematic of different aDBS control strategies. (A) Depiction of a dual-threshold control policy. The upper threshold is depicted by the solid red line and the lower threshold by the dashed red line. The observed beta power is shown in blue. (B) Stimulation intensity is shown in red, adapting within I_min_ and I_max_ in response to the observed beta power relative to two thresholds. A slower ramp rate is used. (C) Depiction of a single-threshold control policy. The single threshold is depicted by the solid red line. The observed beta power is shown in blue. (D) Stimulation intensity is shown in red, adapting between I_min_ and I_max_ in response to the observed beta power relative to the one threshold. A rapid ramp rate is used as stimulation intensity oscillates between I_min_ and I_max_.


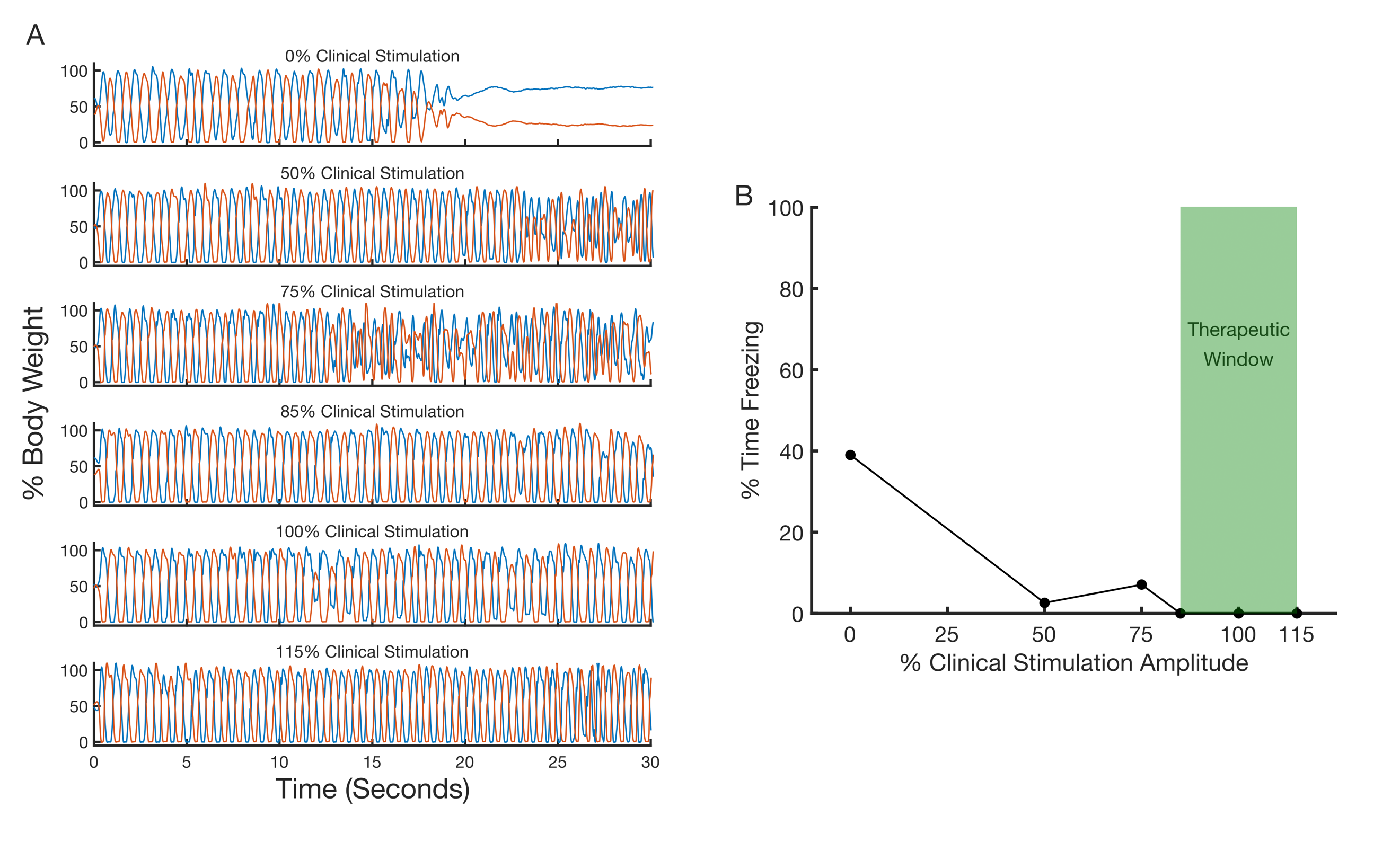


Supplementary Figure 2. (A) Example of stepping behavior at different stimulation intensities. Red and blue traces represent the percent body weight for the left and right foot as the patient alternatively steps in place on two force plates. Rhythmic stepping is reflecting by the alternating oscillation between 0 and 100% bodyweight between the left and right foot as the patient alternates stepping. Freezes are captured when the patient is unable to lift the foot off the plate. (B) The observed percent time freezing across the different stimulation amplitudes with the observed therapeutic window highlighted in light green.
